# Using research findings in my everyday practice: what is good evidence, where do I look, and how can I use it?

**Published:** 2023-01-30

**Authors:** Victor H Hu, Jagadesh C Reddy, Dhivya Rauniyar, Elmien Wolvaardt

**Affiliations:** Assistant Clinical Professor: International Centre for Eye Health (ICEH) LSHTM, and Consultant Ophthalmologist, Mid Cheshire NHS Hospitals, UK.; Founder and Managing Director: Pristine Eye Hospitals Hyderabad, India.; Senior Optometrist: Pristine Eye Hospitals Hyderabad, India.; Editor-in-Chief: *Community Eye Health Journal*, ICEH, LSHTM, London, UK.

## Abstract

The findings from research studies and best practice guidelines should form the foundation of eye care delivery.

As a health care practitioner, you are not best serving your patients if you make decisions based only on your experience and what you learnt during training – especially if you trained some time ago! Although both these sources of learning are valuable, they are not enough. Modern health practitioners are expected to stay up to date with the latest knowledge relevant to their field and to practice evidence-based medicine.

Evidence-based medicine is about using the best available evidence, combined with your own clinical expertise, to make decisions about a patient's health care that respect their values and expectations. But what is the best available evidence, and how can you find it?

If you consider that thousands of research articles are published in eye care journals every year, and that many of them charge high fees for access, it's no surprise that staying up to date with all the latest research in your field is a challenge for most people.

Instead, you may find yourself looking for evidence to answer a specific question. For example, say that you've heard about the potential of collagen crosslinking to prevent the progression of keratoconus, and you want to know whether to start using it. What is the evidence that it works, and that it is safe?

**Figure 1:**
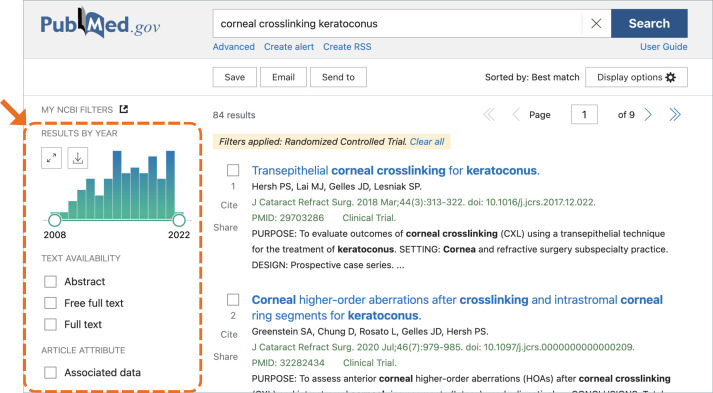
Search results on Pubmed: www.pubmed.gov

## Levels of evidence

Before you start looking for evidence, it is helpful to remind yourself of the different levels of evidence (see panel). Strong sources of evidence, such as systematic reviews, allow you to be more confident in the decisions you make; however, when such evidence is not available, it is useful to know what other types of evidence to look for.

## Finding relevant research

PubMed (www.pubmed.gov) is a large, open access (i.e., free of charge), online database which contains many of the medical research studies which are conducted around the world. Because it is free, and comprehensive, it is a useful starting point when looking for studies on a particular topic.

Another good reason for using PubMed, is that the website makes it easy to filter search results in several useful ways.

For example, try typing the keywords “corneal crosslinking keratoconus” into the search box on the PubMed home page. This produces over 1,800 results. It would be very difficult for an individual clinician to go through all of these before deciding whether to start performing crosslinking.

Did you notice the panel highlighted on the left of the search results in Figure 1? These are options for limiting or ‘filtering’ the results by year, by the availability of the text, article attributes, article type, and so on.

**Figure 2:**
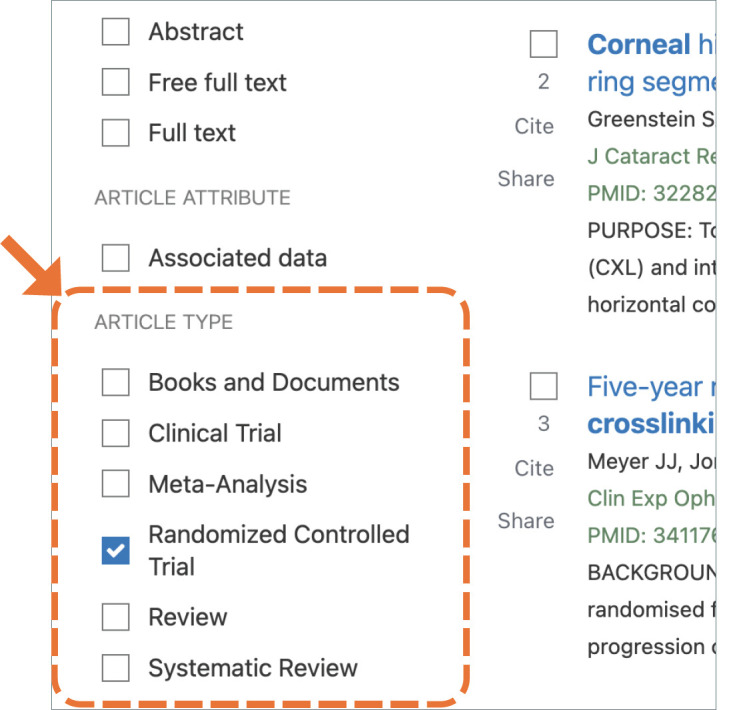
Selecting article type

Referring to the levels of evidence in the panel, and based on how much time we have available, we could decide to limit the PubMed search to randomised controlled trials, which provide a strong level of evidence. To do this, look further down the panel (see Figure 2) and tick the “Randomized Controlled Trial” box under “Article type.” This produces only 85 results. If we limit the results to meta-analysis (a statistical analysis of the results produced by several studies) by ticking that box instead, there are just 23 results for us to evaluate and draw conclusions from.

Looking at well conducted systematic reviews and/or meta-analyses can save a lot of time compared to reading individual studies on a particular area. The Cochrane Library provides some of the highest quality and most trusted reviews available and it is always worthwhile to see if they have done a review on a particular topic: visit www.cochranelibrary.com.

## Good practice guidelines

Despite having access to new online tools such as PubMed, it can still be a challenge to answer all the different questions you face every day by searching for research publications. A practical alternative for busy eye care workers is to use trustworthy, best-evidence clinical practice guidelines.^1^ These are drawn up by teams of people with research experience and knowledge of the area being addressed, who have looked through all the research evidence themselves in a systematic manner. They weigh up all the evidence and come to a balanced judgement on the outcome and what it means for clinical practice. Examples of such guidelines include guidelines from the National Institute of Health and Care Excellence (NICE) in the UK,^2^ the Preferred Practice Patterns from the American Academy of Ophthalmology,^3^ and many others, including disease-specific international societies. It is also important to look at national guidelines which have been drawn up in a particular country. You may even decide to help draw up suitable guidelines for your country or region; these would consider the needs of the local population, the skills of local health workers, and the availability of personnel, equipment, and medicines. The AGREE reporting checklist offers guidance that can help clinicians to evaluate whether a guideline is of high quality or not. It is equally valuable when drawing up clinical guidance.^4^

## Incorporating evidence into everyday practice

The findings from research studies and best practice guidelines should form the foundation of eye care delivery. Alongside this, clinical experience and expertise also form very important aspects of good eye care. Experienced and able clinicians will use evidence in their work but will understand the situation of a particular patient (their medical and social history, risks for that patient, likely adherence to treatment, and so on), what is feasible/realistic in a particular health care context, and where there are gaps in the evidence. Another very important factor to consider is what patients themselves prefer once they have had the different options clearly and coherently explained to them. Practicing medicine is an art as well as a science, and it is important to personalise the management approach for each patient.

**Levels of evidence**

The evidence in this list is arranged from strongest to weakest. Note that each level can be of high or low quality and have a high or low risk of bias or confounding.

1. **Systematic review of randomised controlled trials.** Systematic reviews look at all the studies that have been done on a specific health problem, selecting and assessing them using rigorous, standardised methods. It may include a meta-analysis, which is a statistical analysis of the quantitative results of the studies included in the systematic review. **Meta-analyses** can provide a more precise estimate of an effect than is possible by looking at individual studies.
2. **Randomised controlled trial (RCT).** Participants in the study are randomly allocated into groups, usually to receive or not receive an experimental treatment or intervention. The random allocation helps to ensure a fair comparison (see article 5: Good Research)
3. **Systematic review of cohort or case-control studies.**
4. **Cohort study.** This usually involves many study participants who are observed over a long period (commonly years). The onset of a particular disease (e.g., cancer) can then be compared between people with different levels of exposure (e.g., number of cigarettes smoked).
5. **Case-control study.** People who have a disease (cases, e.g., those with cancer) are compared to a similar group of people (e.g., same age, sex, and socioeconomic level) who don't have the disease (controls). Researchers then work out the level of exposure in the past (e.g., number of cigarettes smoked) and compare them between the two groups.
6. **Case series or case reports.** A single report, or a series of reports, involving patients with a particular disease and who may have been given a similar treatment.
7. **Expert opinion.** This is used where research studies haven't been done on a particular area and people who have experience or expertise on a particular area say what their opinion is.

*Please see the references for more detailed definitions.*
